# Elements of Professional Success

**Published:** 1863-03

**Authors:** B. P. Aydelott


### DENTAL REGISTER OF THE WEST

VOL. XVII.] MARCH, 1863. [NO. 3.

### Original Essays and Communications.

### ELEMENTS OF PROFESSIONAL SUCCESS.

(An Address Delivered at Commencement of the Ohio College of Dental Surgery, February 19th, 1863.)

BY B. P. AYDELOTT, M. D., D. D.

*President of the Board of Trustees.*

THE present age is one of rapidly increasing intelligence, of stirring events, and of unparalleled progress. We must catch its spirit, aim at its objects and heartily throw ourselves into its grand movement, or lose all influence, effect nothing, be nothing. There is no lagging behind in the great social march; not to keep up is to be lost—*lost.*

It becomes, therefore, a most important question, and especially to you, my young friends, who are just about to join the ranks of that vast moving caravan—the living, busy, excited, actual world:—What ought I to be and to do in the position before me? In other words, What are the elements of professional success? And it is to this most momentous question I now invite your serious attention.

I would speak to you as an aged man to those much, very much his juniors; as an old physician to candidates for the responsibilities and honors of one department of the widely comprehensive, noble profession of medicine; as the legally constituted Head of this State College to its favored Alumni; but, above all, as a Minister of the Lord Jesus Christ to young men called of Providence to do a great work for God, for society, for future generations and for their own highest interests.

What then are the ELEMENTS OF PROFESSIONAL SUCCESS? This is our theme. We answer first:

1st. *Competent Professional and preparatory Attainments.*

Dental Surgery embraces many branches of medical science, in each of which the candidate for practice must be duly qualified. And such qualification implies not merely the study of these several sciences, but such skill also in the different manipulations for their practical application as will render the candidate for public patronage competent to manage the cases that, at the very outset of his career, may be entrusted to him.

To acquire this knowledge faithful attendance upon the instructions given in some regular College of Dental Surgery will be found the best means.

But, in addition to these public teachings, each student ought to enter himself as a private pupil in the office of some established practitioner. Much of knowledge, of experience, and of professional skill may be acquired under these private instructions that no public school can impart. So important, indeed, is this latter means of improvement that, in our Medical Colleges, each student who matriculates must be at the same time a private pupil under the care of some regular physician.

But this is not all even of the medical education which you ought to seek.

The human system is, in itself, a unit—strictly a unit—each part of which, both in health and in sickness, sympathizes with every other. Hence no one organ or portion can be long or seriously affected, without involving many others, and, sooner or later, the whole system.

It follows, therefore, that diseases generally in their causes, their courses and their consequences, can not be thoroughly understood and successfully treated without more or less acquaintance with the whole circle of medical science. Hence, at least one course of lectures in some regular Medical College is of high importance to each student of Dental Surgery; or, where this advantage can not be enjoyed, the candidate for the dental degree ought to have carefully studied at least one good system of general Anatomy, one of Physiology, one of the Theory and Practice of Medicine, one of Surgery, one of Materia Medica, one of the Diseases of Women and Children, and one of Forensic Medicine. It is only by such a general knowledge of medical science that any one can be duly qualified for the practice of any particular branch of the healing art, whether it be that of the Oculist, the Aurist, the Surgeon, the Obstetrician, or the Dentist.

But merely medical attainments are not all that is now demanded of you for professional success.

The public mind of this day has become so enlarged and elevated by the general diffusion of education, that every professional man is now expected to have received also a liberal, preparatory education. He can not rightfully take and maintain a respectable position in good society, or in the ranks of the Profession, without such attainments. His conversation must show him to be a man liberally educated, if he would reach the honorable standing, and wield the influence which he ought to possess.

Let it be considered also that the community frequently requires its educated members, and especially those of professional rank, to be trustees and examiners of its educational institutions of every grade from the Common School up to the University. But no mere professional learning, however thorough, will qualify a man for such positions. He must be liberally educated also.

Once more; a liberal education is the best preparation for professional studies. Besides the valuable knowledge acquired in the course of these preparatory studies, the development and the discipline of mind which they give will greatly aid the professional pupil in his peculiar studies. His intellectual tools (if we may be allowed the expression) are all in good order, and of keen edge, and he has acquired also a skill to use them.

But what is of still more importance, a liberally educated man possesses that which no imperfectly educated man can possibly have—a talent of priceless worth and one absolutely essential to the highest success; we mean the power to bring all his faculties at once to bear with undivided, profound attention upon any subject which he may wish to master. He who has acquired this capacity of *mental concentration* is already master of the field. He has little to do but to gather the spoils of victory.

But where may such thorough, preparatory training be best acquired? We mean with the greatest economy of time, money and strength? We answer, most decidedly, in our Colleges of the Arts, as they are usually termed, as Yale, Miami, Wabash. And hence, while it is true that a John Hunter, a Washington, and a Franklin may have actually attained to the highest usefulness and distinction without these preparatory advantages, yet it is a fact also that the great body of professional men, and of eminently distinguished men in church and state, master spirits in peace and war, have been thus liberally educated. The experience of all ages has proved this.

The next element of Professional success we would dwell upon is:

2d. *Manliness.*

Few will deny the importance of this attribute of character, and yet what wrong notions are often entertained about it.

Some suppose manliness to be simply a strong, immovable self-confidence, and that altogether irrespective of the grounds on which it may rest. According to this view, one is merely to work himself up into a high opinion of himself, and then go ahead right or wrong; and this they call manliness. But it is in truth only self-conceit, and differs just as much from true manliness as presumption does from a reasonable faith.

Others confound rashness with manliness. It is only necessary for one, in their view, to rush forward, regardless alike of means and of consequences, to show himself a man. But this is nothing more than foolhardiness, *foolhardiness*, and is commonly found without a particle of real manliness.

Others again mistake wilfulness for manliness; and this mistake is easily made, and not easily detected, because wilfulness does often appear so like manliness as to make it exceedingly difficult to discriminate between them. But after all, it is but a *sham;* there is really nothing in common between mere wilfulness and a true manliness.

Wilfulness cries out "I am determined to do this thing! Don't parley with me; I want none of your counsel; hands off; nothing shall hinder me; I will have my way."

Not so manliness. Manliness is indeed very decided and very firm too, but not at all wilful. "I have before me," says manliness, with a calm, clear eye, "I have before me, an object worthy of my efforts; I have counted the cost, and looked well to my means and to the obstacles in my path; I have reasonable grounds of hope; and this I know and feel that the end to be reached is deserving of all I risk."

Such is what we mean by manliness. An individual of this character endeavors, first of all to form a true, impartial estimate of himself, of his capacity, his fitness; he carefully examines also his objects and surveys the ground before him; and being thus convinced of the goodness of his cause, and that there is a rational prospect of success, and being thus assured also that he is called of Providence to the work, he moves forward calmly but energetically; leaves no stone unturned; faithfully employs all proper means to accomplish his undertaking; and hence rarely fails to reach the goal. This is the manly character. And is not such a character deserving our study? Let us then scrutinize somewhat more closely this important quality of manliness.

First, then; a reasonable self-reliance is an essential ingredient in this attribute of manliness. We say *reasonable*, because it is the result of a thorough honest self-scrutiny. The man that knows himself—what he is, what he can do, and what he cannot do, and what he is fit for. Hence he is rationally self-reliant.

Now, without this quality, no man ever yet accomplished anything—certainly nothing great. The self-distrustful man is always weak and wavering and discouraged at every difficulty. He cannot make up his mind till the opportunity is gone; or he seeks to throw his duties over upon others.

I had a partner, at the outset cf my medical career, in the City of New York who was a pitiable example of this want of self-reliance. Take a single illustration of his character out of scores.

He walked one day, a mile or more, to my residence, summoned me from the dinner table, informed me that he had been called out to see a patient at the other side of the city. I asked him to give me the particulars of the case. He did so. It was clearly an instance of Pneumonia Pleuritis. "You know what is the matter with the man," said I. "Certainly," he replied, "it is Pleurisy." "Well, you know also," I added, "what are the indications, and what are the means of fulfilling these." "Y-e-s," he slowly answered, and then, with a pleasant smile and a child-like importunity, he subjoined, "Doctor, won't you please come and see him?"

The fact is, my poor partner was quite an intelligent physician, and a companion of many estimable points; but he had no confidence in himself; he was not man enough ever to make a medical practitioner.

But, again, candor is another ingredient in a true manliness. A manly man can afford to appear just what he is. Hence, he never thinks of practicing any artifices to conceal weaknesses or defects, or to acquire a character for qualities or attainments which he does not possess, or to keep real difficulties out of view, or to mystify others by an imposing, pompous quackery. On the contrary he feels it no disgrace honestly to say when the occasion calls for it, "I do not know this; or I can not do that; or I would be gratified to hear that another had succeeded in giving the relief which I failed to bring."

Further; the manly man has a confidence not to be shaken in *truth*, in *integrity* and in *honorable dealing.*

There always have been in our fallen world, about every man's path, on the right hand and on the left, continued, and at times, very powerful temptations to false, disingenuous conduct and tricky expedients. And, alas! what multitudes of able and otherwise estimable men, when thus sifted like wheat, begin to lose their hold upon the *true* and the *right*, and fall victims to some corrupt policy to which they are thus tempted.

But the manly man nobly breasts all these assaults—because he believes that truth, integrity and honorable dealing are substantial, precious realities; that these are, under God, our best friends; and that relying upon these, however much we may in the mean while suffer we shall at last come off triumphant.

Hence, a coward expediency has no attractions for a manly character; he is not afraid at any moment to risk his all upon principle. He is ready to reply with Luther, when tempted by some friends to an unmanly policy, "*I would rather die with Christ than reign with Cæsar!*"

And to name no more: The manly man will exercise a generous confidence in his fellow men, and especially in his professional brethren.

He does not indeed foolishly suppose that "all is gold that glitters;" but neither does he, on the other hand, look upon every man as a villain. No; his heart assures him that there is such a thing as honesty and trustworthiness in the world; and he is willing to bestow a generous confidence where he has no reason to suspect ill-desert. And such a man is not often deceived, and very rarely to his great injury.

But this is not all: His manifestly generous confidence in others will often have the happiest effect by encouraging and cherishing the growth of a true trustworthiness in many around him, who, perhaps, hitherto had little claim to that virtue.

But besides greatly benefiting his fellow men in this way, the reflex influence of a generous confidence in others is full of blessing to the man himself. Did you ever know, my young friends, a man of dark suspicious temper greatly successful in life, or enjoy much happiness? I never did. And, on the other hand, did you ever know a man of generous confiding spirit greatly fail in life? or be other than an habitually a cheerfal man?

Look back, then, my young hearers, and examine these elements of a manly character—its reasonable self-reliance, its manifest candor, its calm unshaken trust in truth, integrity, and honorable dealing, its generous confidence in others, and do you not see in these that true manliness must be a powerful element of success, because it raises up hearty helpers all around it by promoting the growth of others of just such a character; and because also the world can not help seeing and admiring snch a character wherever it exists? and hence the world will rarely fail to give such a man a generous confidence in return and so greatly enlarge his field and his power to do good to others and get good to himself.

Before quitting this part of our subject let us very briefly add that the possession of manliness, in whatever else we may be deficient, can scarcely fail to insure success.

Look at such a man in a Society, or a Board — either educational, benevolent, or religious—and you may be sure to find his influence all-pervading, governing. And in the various walks of life too—however humble his position—the world around him will, sooner or later, feel that it has a man among them, a *live man.* His manifest truth, integrity and honorable dealing, combined with a wise energy, will outweigh every thing that may be brought against him; and give him a power that nothing else can impart, nothing long resist.

Yes, my young friends, this virtue of manliness will make up for great defects in talents, learning, genius and fortune. Like charity, it will cover a multitude of sins. But without manliness, however gifted and favored an individual may be in all other respects, he can accomplish little. A most striking illustration of the value of this attribute of manliness we have in the character and career of Washington. He had not great powers of intellect; to brilliancy he made no pretensions whatever, and his education was very limited; but he possessed, to a remarkable degree, that calm, strong, unshaken manliness which inspired confidence and respect in all around him; raised up means of success and trustworthy men—helpers—at every step; gave him a full knowledge of his position and all his resources; and kept him cool, steady and firm in the path of principle, till, through difficulties and dangers which few ever meet be accomplished under God's blessing, greater results than almost any other mere man ever achieved.

But let us endeavor to throw yet more light upon this important point.

Attempts have been made to draw a *comparison* between Washington and Napoleon. A *contrast*, we think, could be much more easily delineated. For scarcely ever were two so distinguished men more unlike.

What Washington was, we have endeavored above very briefly to show; let us now look for a moment at Napoleon. He had vast intellectual powers; for brilliancy of genius few ever excelled him; and his education at the school of Brienne was thorough, and admirably adapted to fit him for the career before him. He had an iron will, also, before which friends and foes must alike yield; it was scarcely possible to resist one so determined and set in his way. His courage, too, like indeed that of Washington, never faltered, but unlike the latter, it often ran into audacity—at times a *sublime audacity.* Indeed to this latter quality he often owed his success. He was ready to risk all at any moment upon the cast of a die. He would attempt what no one supposed that a man in his senses could think of; and hence he here often succeeded, because others were totally unprepared for such an onset.

But with all his vast intellectual powers and his incomparable genius, and his admirable educational training, and his iron will and daring courage, and after years of unparalleled success, often against the most fearful odds, he was at last irretrievably overwhelmed with disasters, and died in circumstances so deeply humiliating that even those who most feared and abhorred him could not but pity him.

But what was the cause of this miserable wreck of all his abilities, his attainments, his vast aims, and the wonders he actually accomplished? Why has it all vanished like the gorgeous fabric of a dream? It was Napoleon's want of true manliness—that for which Washington was pre-eminent and which mainly sustained him and bore him onward to the loftiest pinnacle of human grandeur. Yes, here was the terrible secret—Napoleon had *little or no faith in truth, integrity and honorable dealing.* Hence, when calmly and honestly judged in the light of God's law—the only standard of pure eternal rectitude—Napoleon must be pronounced—what indeed most of the world's heroes have usually been—little other than *a magnificent villian!*

We have now enumerated and very briefly dwelt upon certain elements of professional success; and we suppose that none will deny their importance, indeed their necessity.

Certainly a man not competent in point of knowledge and skill for the practice of a profession, and without that manliness which alone can sustain him under the duties and trials that must necessarily come upon him; certainly such a man can not succeed, indeed he ought not. He may, perhaps, by unworthy arts, or under peculiarly favorable circumstances, obtain temporary reputation, but he can not achieve permanent respectability and usefulness.

Here, however, we may be met with some important questions, which it would be improper on such an occasion to pass over.

As, first, is there not much in the present state of the world to hurry young men into the struggle of life without any adequate education—preparatory or professional? And secondly, when once embarked in the world are there not temptations, on the right hand and on the left, to draw them out of the plain path of truth, integrity and honorable dealing, and lead them into doubtful expedients and dishonorable practices and downright quackery, and, in the general, to a course of conduct unbecoming an intelligent, liberal, lofty, philanthropic profession? And, lastly, in view of these temptations how is it possible for fallen, feeble human nature to resist?—always to stand upright?—and hold on the even tenor of its way, notwithstanding every allurement, every assault?

Certainly the objections here are all real, and they are very moderately stated too. If, indeed, by ignoring them, we could annihilate them, we might wisely close our eyes against them. But it is folly so to do. We must manfully meet them; and therefore it is our wisdom, if we can, to be fully prepared for the trial.

But after all may not the best training preparatory and professional, which we can make—and may not mere manliness too—all go down in the struggle, the great battle of life? Or, is there a way, and are there means by which we may pass unscathed through this perilous ordeal, and come out of the furnace without even the smell of fire upon our garments?

We believe, my young friends, there is such a way and that there are such means. Indeed, without this confidence we would not have dared to stand here on the present occasion, because we should only mock your hopes by spreading before you a most desirable, noble prospect, with the painful conviction that you could never realize it. We say, then, thirdly and finally,

III. *True Christian character* will ensure to every well-instructed, manly candidate an ample measure of professional success and honor.

How this character is to be reached the gospel so clearly makes known to us, that "The wayfaring man, though a fool, need not err therein." The gospel does indeed honestly spread out before us all our evils and difficulties and dangers—our guilty, corrupt, ruined state, through sin; but it graciously points us also to an Almighty Deliverer, the Lord Jesus Christ, the Divine incarnate Savior, who, by the sacrifice of himself on the cross, freely offers us redemption not only from the guilt of sin, but from the darkness and the bondage of sin.

He who embraces these gracious proffers of the gospel at once exchanges all his guilt and all his guilty fears for joy and peace in believing, and he comes from under the debasing bondage of sin and Satan into the holy, happy liberty of God's children. In this way is he brought into harmony with himself, and into a state of reconciliation with God—the Sovereign Ruler and Disposer of all events. Hence, he now sees plainly before him the path of duty leading onward and upward, onward and upward, to a glorious immortality; and he knows that in the meanwhile, too, "all things," even the darkest trials here below, "work together for good to them who love God."

Now is not such a man in the best possible condition and circumstances for success? He has peace within, and therefore with nothing to divide and distract his attention, all his powers, intellectual, moral, physical, are brought into full, cordial, co-operation for the accomplishment of the high and blessed destiny that awaits him. He has also that trust in God which will sustain him in every trial, nerve his arm for every duty and carry him safely and successfully through every difficulty and danger. In a word, he is master of himself and so he is a *whole man*, and still more he has *God for his friend.*

Now what can harm such a man? And what can he not accomplish? He is prepared for all that is before him in the great contest of life; and he will most assuredly be brought off "conqueror, yea, more than conqueror, through him that loved him and died for him."

And now, my young friends, I have utterly failed in all I have said at this time if I have not fastened upon your minds two unspeakably important lessons—first,

1. That you have a great work before you—one that demands and richly deserves close study, patient labor, and the faithful application of all your time and of all the powers of your nature—body, soul and spirit.

In a word; you must henceforth be true professional men, and to be that, in the highest sense, you must be true Christians. And secondly,

2. While laboring to secure such a character with all diligence, you must look upward, upward, at every step; you must have faith in God through our Lord Jesus Christ.

Be assured, my young friends, this life of honest, patient, cheerful effort, and of humble, devout dependence is the only true life. It resolves many difficult problems, otherwise totally inscrutable. There is nothing desirable it cannot accomplish.

And be assured also that it is as impossible for such a life to fail in its great end, as it is to shake the pillars of the Eternal Throne. "If God be for us, who can be against us?"

And to sum up our whole instructions in a few brief sentences. EFFORT AND DEPENDENCE, EFFORT AND DEPENDENCE is the grand secret of life. Every true Christian, whatever his position in the world, realizes this. EFFORT AND DEPENDENCE, EFFORT AND DEPENDENCE is his wisdom, his strength, his success. With these we can do all things; without these—however else gifted and favored—we shall miserably perish.

